# Morphological and Mechanical Characterization of Extracellular Vesicles and Parent Human Synoviocytes under Physiological and Inflammatory Conditions

**DOI:** 10.3390/ijms232113201

**Published:** 2022-10-30

**Authors:** Samira Filali, Nesrine Darragi-Raies, Layth Ben-Trad, Agnès Piednoir, Saw-See Hong, Fabrice Pirot, Ahmed Landoulsi, Agnès Girard-Egrot, Thierry Granjon, Ofelia Maniti, Pierre Miossec, Ana-Maria Trunfio-Sfarghiu

**Affiliations:** 1 Immunogenomics and Inflammation Research Unit EA 4130, Department of Immunology and Rheumatology, Edouard Herriot Hospital, Hospices Civils de Lyon, University of Lyon, 69007 Lyon, France; sfilali@15-20.fr; 2 Laboratory of Research and Development of Industrial Galenic Pharmacy and Laboratory of Tissue Biology and Therapeutic Engineering UMR-CNRS 5305, Pharmacy Department, FRIPHARM Platform, Edouard Herriot Hospital, Hospices Civils de Lyon, University of Lyon, 69007 Lyon, France; fabrice.pirot@chu-lyon.fr; 3 Laboratory of Contact and Structural Mechanics, University of Lyon, CNRS, INSA Lyon, UMR5259, Villeurbanne, 69100 Lyon, France; darraginesrine@outlook.fr (N.D.-R.); layth.ben-trad@univ-lyon1.fr (L.B.-T.); 4 Laboratory of Risques Liés aux Stress Environnementaux: Lutte et Prévention, Faculty of Sciences of Bizerte, Université of Carthage, Zarzouna 1054, Tunisia; ahmed_landoulsi@yahoo.fr; 5 Institute de Chimie et Biochimie Moléculaires et Supramoléculaires, ICBMS, UMR 5246 CNRS, University of Lyon, 69622 Lyon, France; agnes.girard-egrot@univ-lyon1.fr (A.G.-E.); thierry.granjon@univ-lyon1.fr (T.G.); ofelia.maniti@univ-lyon1.fr (O.M.); 6 Institut Multidisciplinaire de Biochimie des Lipides, 69621 Villeurbanne, France; 7 ILM, UMR 5506 CNRS, University of Lyon, 69621 Villeurbanne, France; agnes.piednoir@univ-lyon1.fr; 8 UMR 754 UCBL-INRA-EPHE, Unit of Viral Infections and Comparative Pathology, 69366 Lyon, France; saw-see.hong@univ-lyon1.fr

**Keywords:** extracellular vesicles, synoviocytes, synovial fluid, osteoarthritis, rhumatoid arthritis, inflammation

## Abstract

The morphology of fibroblast-like synoviocytes (FLS) issued from the synovial fluid (SF) of patients suffering from osteoarthritis (OA), rheumatoid arthritis (RA), or from healthy subjects (H), as well as the ultrastructure and mechanical properties of the FLS-secreted extracellular vesicles (EV), were analyzed by confocal microscopy, transmission electron microscopy, atomic force microscopy, and tribological tests. EV released under healthy conditions were constituted of several lipid bilayers surrounding a viscous inner core. This “gel-in” vesicular structure ensured high mechanical resistance of single vesicles and good tribological properties of the lubricant. RA, and to a lesser extent OA, synovial vesicles had altered morphology, corresponding to a “gel-out” situation with vesicles surrounded by a viscous gel, poor mechanical resistance, and poor lubricating qualities. When subjected to inflammatory conditions, healthy cells developed phenotypes similar to that of RA samples, which reinforces the importance of inflammatory processes in the loss of lubricating properties of SF.

## 1. Introduction

The synovial joint allows the realization of multiple, complex, and precise movements over a period of more than 80 years, surpassing any device created by humans. These performances are due to the “cartilage-synovial liquid-cartilage” tribological system, which supports and redistributes the forces in the articular contacts and guarantees minimum friction and maximum shock absorption [1,2,3]. The synovial fluid (SF) is a bio-fluid present in the synovial joint space [4,5,6]. It is an ultra-filtrate from plasma to which are added elements of cell synthesis from cells present in the synovial membrane, the membrane which surrounds the synovial joint and, together with the infrapatellar fat pad, constitutes an anatomo-functional unit [7,8]. This last, unlike cartilage, is richly innervated and is potentially involved in the onset of articular diseases and pain [9]. Pain in osteoarticular diseases is of multifactorial origin, involving both peripheral and central mechanisms [10,11,12], notably irritation of sensory nerve endings from osteophytes and synovial inflammation, with release of chemical mediators, which in turn will sensitize the primary afferent nerves. 

Two types of cells are present in the synovial membrane. The first ones are immune cells called macrophages or type A synoviocytes. The second type are fibroblast-like synoviocytes (FLS), or type B synoviocytes, which are secretory cells responsible for the production of elements of SF such as hyaluronic acid or lubricin [13,14,15,16,17]. FLS adopt a filamentary elongated shape to form pseudopods at the cell periphery. The FLS endoplasmic reticulum is well developed. FLS play a major role in joint pathologies by the induction of the serial reactions of the immune system such as the overproduction of cytokines and chemokines [18,19,20].

A deficient lubrication contributes to the erosion of the cartilage surfaces. Depending on the quality of the synovial fluid, direct contact between the cartilaginous surfaces can cause bone wear and both chronic and acute pain [5,21,22,23]. The number of people suffering from joint diseases is constantly increasing worldwide. The most common joint diseases include osteoarthritis (OA), a degenerative and debilitating disease [24,25], and rheumatoid arthritis (RA), a chronic inflammatory autoimmune disease [26,27,28,29]. Both pathologies can be caused by exogenous or endogenous sources, such as genetics or hormones [30]. In addition, joint pathologies involve pro-inflammatory interleukins [31,32]. Human IL-17, for instance, a proinflammatory cytokine identified in 1995 as a product of activated T cells, is involved in the pathogenesis of RA and many other autoimmune and inflammatory diseases (reviewed in [33]) and constitutes an interesting therapeutic target [34]. Local and systemic effects of IL-17 in joint inflammation are often associated with other inflammatory factors such as TNF-α [35]. The combination of IL-17 and TNF-α induces the expression of 9803 inflammatory genes [36] with an additive synergistic effect [37]. Activation of the ubiquitous IL-17 receptors in different immune cells causes amplification of inflammation in the joint space and inhibition of regulatory mediators [38,39]. IL-17 and TNF-α also have synergistic effects on the production of other cytokines in synoviocytes [40]. Several studies have also revealed that IL-17 is involved in OA pathogenesis and may constitute an interesting target [41,42,43], although studies on OA pathophysiology have focused more on cartilage degeneration and osteophytes, rather than on the inflamed and thickened synovium. FLS produce a series of pro-inflammatory regulators, which are positively associated with the clinical symptoms of OA, such as inflammatory pain, joint swelling, and disease development [20]. 

Alteration of the SF composition was noted in the case of pathologies such as OA and RA [44], with a decrease in the hyaluronic acid concentration (0.7–1.1 mg/mL for OA and 0.8–1.5 mg/mL for RA) and a remarkable increase in protein concentration (29–39 mg/mL for OA and 36–45 mg/mL for RA) and phospholipids (0.2–0.3 for OA and 1.5–3.7 mg/mL for RA) compared to healthy synovial fluid (1–4 mg/mL hyaluronic acid, 15–25 mg/mL proteins, and 0.1 mg/mL phospholipids). Changes in lipid composition or in lipid concentration in the synovial fluid also have important consequences for the bio-lubrication properties of the SF [45,46,47] and for cartilage wear [48]. The SF is discontinuous and contains microvesicular structures coated with lipid/water multilayers, called synovial vesicles. In a previous study, we analyzed the ultrastructure of vesicles present in healthy and pathological SF [49]. The phospholipid concentration in SF was found to increase in pathological contexts, but the proportion of phospholipids relative to the overall lipids decreased. Large multilamellar microvesicles filled with glycoprotein gel were observed by transmission electron microscopy in healthy subjects, which were proposed as the signature of healthy SF. In RA and OA samples this structure was severely altered, with a predominance of small vesicles surrounded by a glycoproteic gel. Changes in phospholipid proportion and chemical composition thus induced structural alteration both in SF of the OA and RA patients, to which dysfunctions in lubricating properties of SF were in turn attributed [49]. 

Extracellular vesicles (EV) are submicron, membrane-derived vesicles secreted by all human cell types [50,51]. The term “extracellular vesicles” is largely used to describe exosomes, microvesicles, and apoptotic vesicles that differ in size, biogenesis, and biomolecular composition, although there is no clear-cut test to classify each sub-group of vesicles. Exosome-like EV (small-sized vesicles of endosomal origin) are present in SF and have been attributed roles in the development and progression of joint pathologies such as OA and RA [52,53,54,55]. In this study, we focus on synovial vesicles, a particular type of extracellular vesicles synthesized by FLS present in SF, which have an important part in the lubrication of the joint [56,57,58,59].

A described above, SF is an exudate from plasma supplemented by active synthesis from FLS which secrete synovial vesicles. Are the alterations evidenced in OA and RA SF extracts [49] a consequence of plasma alterations from inflammatory or metabolic reactions or is the intrinsic synthesis of the synovial vesicles modified? In the present study, we dissociated synoviocyte synthesis from plasma lipid exchange to respond to this question. 

Therefore, we developed human FLS cultures issued from OA and RA patients or from patients undergoing surgery for mechanical issues, considered as healthy from an osteoarticular point of view, grown either in normal medium or in a medium supplemented with inflammatory cytokines. The morphology of FLS, as well as the ultrastructure and the mechanical properties of the synthesized EV, were analyzed and compared.

## 2. Results

### 2.1. Choice of the Optimal Analysis Conditions for FLS and Secreted EV

#### 2.1.1. Selection of the Appropriate Time-Point to Perform Morphological and Mechanical Characterization

**Figure 1:**
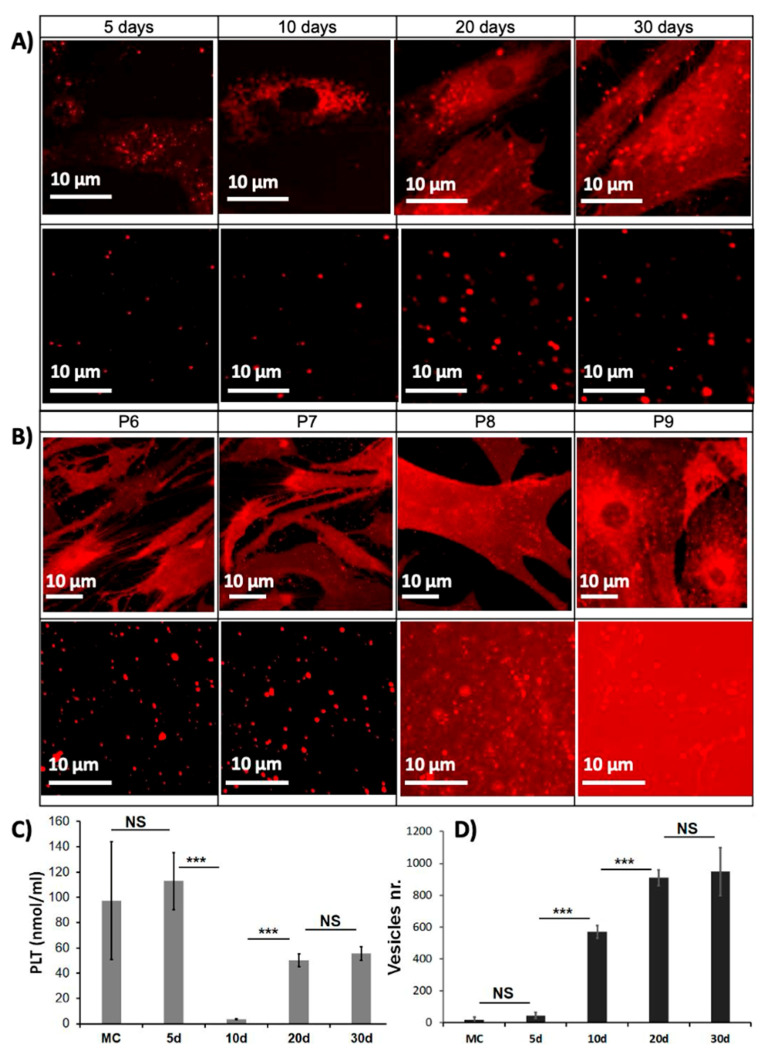
Selection of the most appropriate conditions for the structural and mechanical characterization. (**A**) Cells (**top**) and supernatants (**bottom**) confocal micrographs at 5, 10, 20, and 30 days of cell culture (X63 immersion oil microscope objective) after passage 6. (**B**) Cells (**top**) and supernatants (**bottom**) micrographs from passage 6 to 9 each after 20 days. Labeling of the different components was done by lipophilic fluorescent label “DiI” (λex~549 nm). (**C**) PLT concentration (µg/mL) in the supernatant and (**D**) number of vesicles per image as a function of cell culture day. MC, medium of culture. Bars represent mean ± SD. *** *p* < 0.0001, and NS non-significant.

In a first step, to determine the appropriate time-point for cell and lipid vesicle structural and mechanical characterization, FLS at passage 6 were analyzed after 5 days, 10 days, 20 days, and 30 days under non-inflammatory conditions (Figure 1A). Cells were stained with DiI and visualized by confocal microscopy (Figure 1A, top panels). After 5 days of culture, cells showed a low fluorescence intensity, indicating that the DiI was not well embedded in the cells. After 10 days, a better incorporation of the fluorescent marker in cells was observed, but the fluorescence was mainly localized around the nucleus. After 20 days, FLS were well marked and the fluorescence was distributed all over the cell. This efficient labeling was also observed 30 days after P6, but cell density became important and cells started to overlap.

To monitor cell production of vesicles, vesicles from the supernatants were visualized (Figure 1A, bottom panels) and the PLT in the supernatant was quantified using the Steward method (Figure 1C) and the number of particles was counted using the ImageJ function Analyse particles

After 5 days of culture, vesicles with diameters ranging from 0.2 to 0.8 µm were uniformly distributed on the entire glass slide (Figure 1A, bottom), but their number was low—less than 100 vesicles per image (Figure 1D). Phospholipid concentration in the supernatant at this time-point was not significantly different from that determined in the cell culture medium (Figure 1C). Indeed, fetal bovine serum-supplemented medium is known to contain vesicles and phospholipids from FBS. These results suggest that after 5 days the cellular metabolism of FLS was not active enough to maintain a good production of synovial vesicles. Therefore, the observed vesicles were mainly coming from the FBS- supplemented DMEM. In addition to the observed vesicles, some phospholipid structures were present in the supernatant, but their size was below microscope resolution.

After 10 days, vesicles were much bigger, with diameters ranging between 0.6 and 2 µm, but their number was significantly increased to 600 vesicles per image. Moreover, they were distributed heterogeneously in the sample. The phospholipid concentration was rather low in comparison with the DMEM-FBS medium that served as control and with the 5-day cell culture medium. We can therefore suggest that after 10 days of culture, cells consumed almost all of the phospholipids present in the culture medium and started to produce their own lipid vesicles.

After 20 days, the number of lipid vesicles further increased to 900 vesicles per image (Figure 1D), with a homogeneous distribution over the entire surface of the glass slide. PLT concentration significantly increased in comparison with the concentration determined at 10 days (Figure 1C). These findings suggested that after 20 days of cell culture, the cellular metabolism was active and a sufficient quantity of vesicles, specifically issued from FLS, was obtained to allow structural and mechanical characterizations.

At 30 days, there was no notable difference in either the number of vesicles produced, or in the phospholipid concentration in comparison to results obtained after 20 days of culture. 

Altogether, these results indicated that the optimum time-point for synoviocyte and synovial EV analysis was after 20 days of culture. All subsequent experiments were carried out at this time-point. 

#### 2.1.2. Choice of the Appropriate Number of Cell Passages

As described in the literature, in order to obtain relevant synoviocyte cultures, at least four passages in the appropriate medium are required [39]. Therefore, synoviocytes are usually analyzed at a number of passages between 4 and 8. To choose the most relevant number of passages for the analysis of the EV produced, confocal micrographs from the sequential passages from 6 to 9 for the same synoviocyte culture were recorded. Figure 1B shows synoviocyte morphology for P6 to 9 (top panels) and vesicles present in the supernatant (bottom panels).

Cells from P6 and P7 had a high number of pseudopods and sustained a good production of lipid vesicles. In contrast, cells from P8 and P9 were spread out, with fewer pseudopods. In terms of synthetized EV, supernatants from P6 and 7 contained well-distinguishable micrometric vesicles (Figure 1B, bottom panels). At P8 and 9, supernatants showed a red background together with bright, large spots, which may correspond to a coexistence of aggregated micrometric vesicles and nanometric vesicles undistinguishable at the microscope resolution. 

In light of these observations, experiments here-on were performed with FLS at P6 followed by 20 days of culture. 

### 2.2. Impact of Disease on FLS and on Synovial Vesicles

To assess the impact of disease on FLS and on the quality of the synthesized synovial EV, a comparison of the structural and tribological characteristics was made between synoviocyte cultures issued from healthy patients and patients with clinically established RA and OA disorders.

#### 2.2.1. Ultrastructural Characterization at Cell Level by Confocal Microscopy

**Figure 2:**
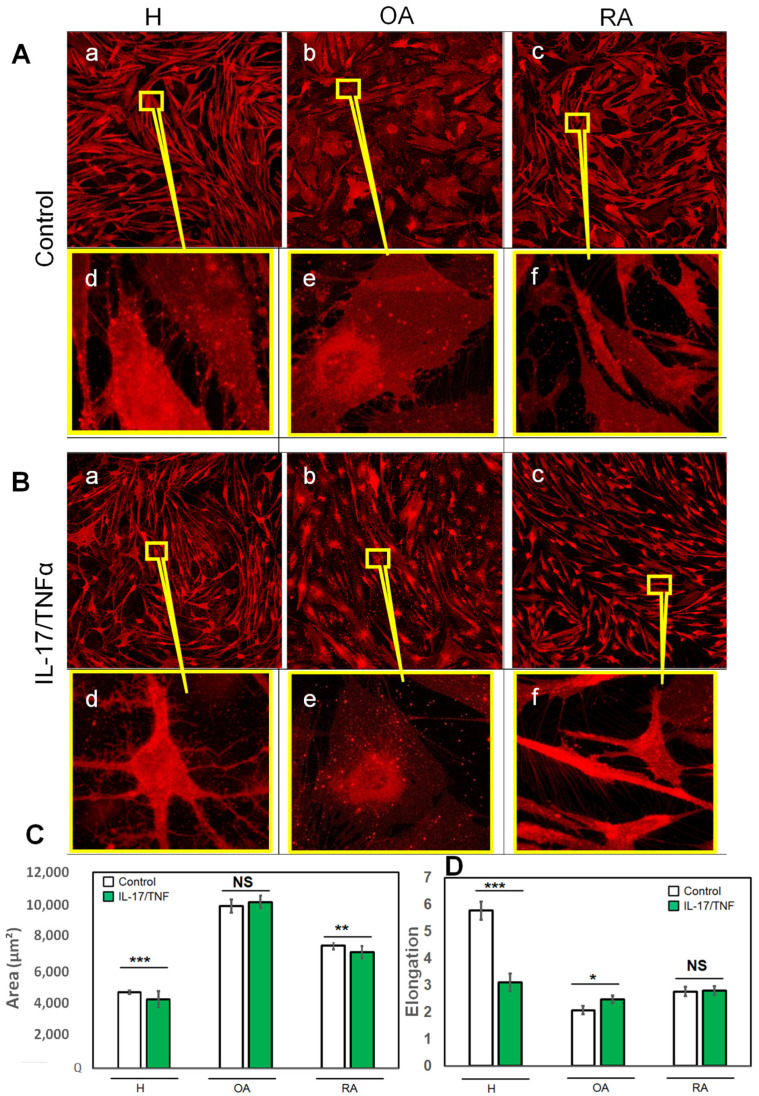
Structural characterization of FLS in the absence (**A**) or presence (**B**) of an inflammatory environment (IL-17: 50 ng/mL, TNF-α: 1 ng/mL). Cells at 20 days of culture after passage P6 were labeled with lipid fluorescent label “DiI” (λex~549 nm) in all cases. Differences in cells morphology in term in terms of shape, spread and number of pseudopodia were observed by confocal microscopy (**a**–**f**). Synoviocyte area (µm^2^) (**C**) and elongation (**D**) in the absence or presence of inflammatory environment. Bars represent mean ± SEM. *** *p* < 0.0001, ** *p* < 0.001 * *p* < 0.01 and NS non-significant.

Figure 2 shows the morphology of FLS cultivated in supplemented DMEM (A) and in the presence of inflammatory factors IL-17 and TNF-α (B). At a large scale (X5 microscope objective), H cells grown in non-inflammatory medium for 20 days (Figure 2(Aa)) had a typical, uniform FLS morphology, with an elongated shape and a homogenous distribution of fluorescence over the cell. OA cells grown for 20 days in non-inflammatory medium (Figure 2(Ab)) were spread out and round shaped. The fluorescence labeling was very intense around the nucleus and low at the periphery. RA cells grown for 20 days in non-inflammatory medium (Figure 2(Ac)) were more elongated than OA cells, but less than H cells. The distribution of fluorescence was homogenous over the cell, like the H control cells.

Quantitative analysis at this magnification (Figure 2C,D) confirmed these observations. The average total area of H control cells determined from the confocal microscopy images was significantly smaller than that of OA and RA cells (Figure 2C, white bars). To take into account the cell shape (round or elongated), an elongation factor was calculated as being the ratio between the maximum and the minimum diameter of the cell (Figure 2D, white bars). This factor was higher for H cells, as they had filamentary shapes, with a value of 6, and drastically decreased to a value of 2 and 2.5 for OA and RA, respectively, the two adopting more rounded shapes. 

At higher magnification (X63 microscope objective), further details in cell morphology were observed. In the absence of inflammatory factors, some pseudopod connections between H cells were formed and micrometric lipid vesicles were observed inside the cells (Figure 2(Ad)). Vesicles were uniformly distributed inside the cell. When compared to H control cells, OA cells formed numerous pseudopods, which occupied the entire peripheral cell area (Figure 2(Ae)). An increase in the number of pseudopods and intercellular connections was also observed for RA cells (Figure 2(Af)). Altogether, OA and RA cells have different morphology patterns. H and RA cells are characterized by elongated cells, with very long filamentous shapes for H, whereas a round cell body is noticed for OA cells.

OA or RA pathological contexts thus alter synoviocyte morphology in terms of cell shape and area, as well as in the number of pseudopods. To see whether such changes are due to the existence of an inflammatory environment, we mimicked pathological inflammatory situations by adding pro-inflammatory cytokines IL-17 and TNF-α in the growth medium (Figure 2B). In the case of H cells, the morphology of the cells was drastically impacted (Figure 2(Ba)). H pleomorphism was illustrated by cells shrinking and forming branched pseudopods covering the cell periphery. In the presence of inflammatory cytokines, the average total area and the elongation factor decreased from 4000 µm^2^ to 3000 µm^2^ and from 6 to 3, respectively (Figure 2C,D, green bars).

**Figure 3:**
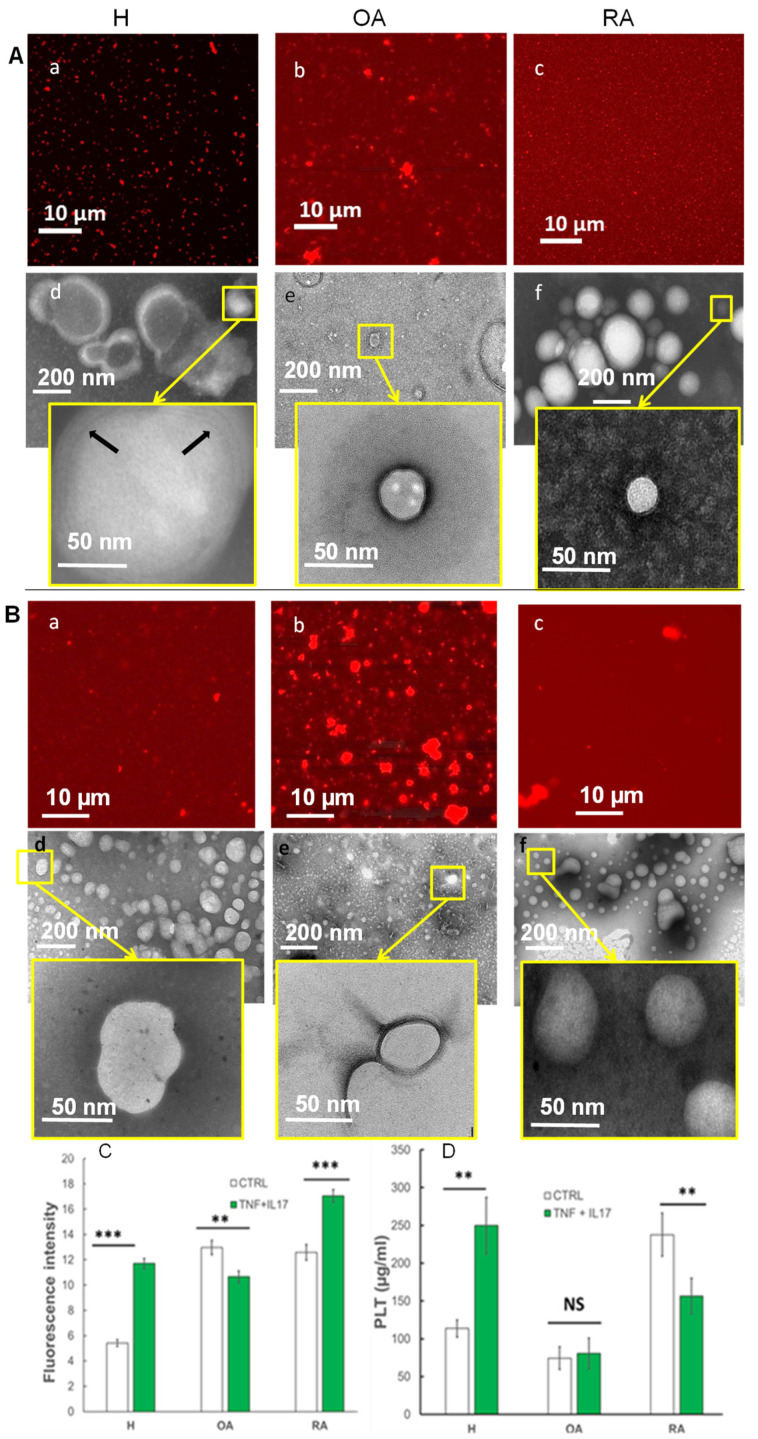
Structural characterization and comparison of supernatants between healthy (H) and pathological (OA and RA) in the absence (**A**) or presence (**B**) of inflammatory environment (IL-17: 50 ng/mL, TNF-α: 1 ng/mL). Differences in vesicular structures were observed by confocal microscopy (**a**–**c**) and transmission electron microscopy (TEM) (**d**–**f**) (20 days of cell culture after cell passage 7). Arrows: Phospholipid multilayers. (**C**) Fluorescence intensity of the image. (**D**) PLT concentration (µg/mL) in the supernatant. Bars represent mean ± SEM; *** *p* < 0.0001, ** *p* < 0.001 and NS non-significant.

In the case of OA grown under inflammatory conditions, the number of inter-cellular pseudopods was reduced with respect to non-inflammatory conditions (Figure 3(Bb,Be)). The average total area of OA cells (10,000 µm^2^) in the presence or absence of cytokines did not show a significant difference (Figure 2C). The elongation factor was higher in the inflammatory medium relative to that of OA under reference conditions (2.8 and 2.4, respectively), attesting to a change in cell morphology towards a more elongated form (Figure 2D). 

RA cells grown with the pro-inflammatory cytokine treatment were smaller, with long pseudopods intensively labeled by the fluorescent probe (Figure 2(Bc,Bf)). Although a significant decrease in the cell area under inflammatory conditions was recorded (Figure 2C), no significant difference in the elongation factor was observed, attesting that, overall, the shape of the cells was unaltered (Figure 2D). Both OA and RA cells were monomorphic, inflammatory conditions inducing a “homogenization” in forms and shapes. In the inflammatory case, the differences in cell morphology (area and elongation) between OA and RA, were still present, with large round-spread OA cells and smaller, more elongated RA cells. (Figure 2C,D). 

#### 2.2.2. Ultrastructural Characterization of Synovial Vesicles under Healthy and Pathological Conditions

Vesicles synthesized in each cell culture condition (H, OA, and RA) were visualized by fluorescence microscopy using a X63 microscope objective (Figure 3, the first and the third line). In the supernatants from H cells, lipid vesicles with micrometric sizes were well separated by a black background (Figure 3(Aa)). The fluorescence intensity of the extra-vesicular background was thus rather low, corresponding to a very low lipid quantity outside the EV (Figure 3C). The concentration of the total phospholipids present in these supernatants was determined by the Stewart method (Figure 3D) and was about 0.1 mg/mL, close to that reported for the healthy synovial fluid samples [44,49]. 

In the presence of pro-inflammatory cytokines, H supernatants (Figure 3(Ba)) showed a highly fluorescent background in the extra-vesicular space making micrometric lipid vesicles less visible. These results were correlated with a very significant increase in the total phospholipid quantity that was 2.5 times higher for H (IL17 + TNF- α) than that of the control H cells. Thus, inflammation alters the production of extracellular vesicles by FLS.

**Figure 4:**
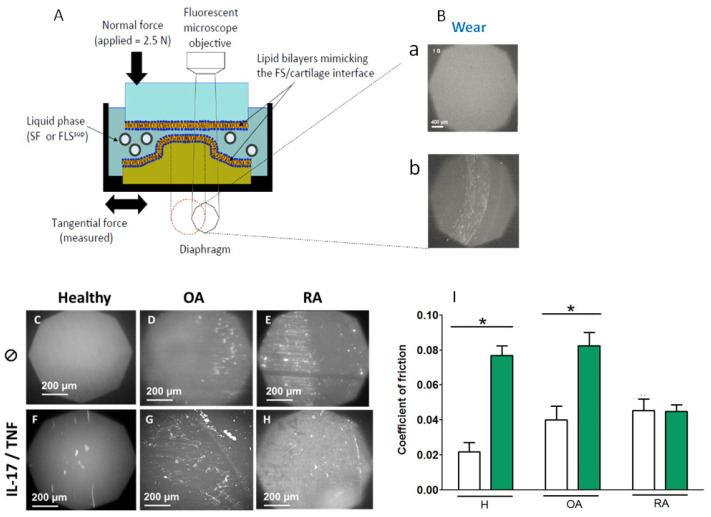
Tribological behavior of vesicle supernatant (H, OA, and RA) after wear and friction tests. (**A**) The principle of functioning of the bio-tribometer apparatus showing the ex vivo contact model. (**B**) Wear: micrographs showing the wear of the lipid interfaces deposited at on the surfaces in contact using white light microscopy (**a**) unworn interface, an even image in the viewing octagon of the microscope (**b**) interface degradations, lighter clusters in the viewing octagon of the microscope. (**C**–**H**) Micrographs of the lipid interfaces wear for vesicle supernatant (H, OA, and RA) on absence and presence of inflammatory environment. (**I**) Coefficient of friction as a function of pathologies in the absence (white bars) or presence (green) of inflammatory environment (IL-17: 50 ng/mL, TNF-α: 1 ng/mL). Error bars represent SD values calculated on at least 3 independent measurements. * *p* < 0.01.

Supernatants of OA cells in the absence or presence of IL-17 and TNF-α (Figure 3(Ab) and Figure 4(Bb)) presented numerous micrometric vesicle clusters situated in an extra-vesicle fluorescent background. A high overall fluorescence was measured in both samples (Figure 3C) which did not correlate with the very low phospholipid concentration measured in these supernatants (Figure 3D). 

For RA supernatants in the absence of inflammatory cytokines (Figure 3(Bc)), we noted the presence of many submicrometer vesicles, visible with a X63 microscope objective, together with a very high fluorescence intensity outside vesicles (Figure 3C) and a quantity of phospholipids about 2.5 times more important than that of H supernatants (Figure 3D). This later finding was consistent with the literature reporting a significant increase of phospholipid concentration in synovial fluid of patients with RA [44]. This means that RA control samples were composed of a more important number of lipid vesicles than that of H samples, that is, very small and dispersed vesicles, below microscope resolution, resulting in an intense red background. The addition of pro-inflammatory cytokines in the medium provoked the formation of clusters of lipid vesicles together with a fluorescent background in the extra-vesicular space much more intense than that of RA supernatants. However, these were not correlated with total phospholipid quantity results which showed a significant decrease in phospholipid concentration in the case of RA (IL17 + TNF-α) compared to that in RA control. 

To sum up, the observation of lipid vesicles with X63 microscope objective demonstrated the presence of micrometric vesicles in the healthy case (H). In contrast, in the case of RA and H in inflammatory conditions, numerous but smaller submicrometric, vesicles were observed, correlating well with an increase in phospholipid concentration. A fluorescent background corresponding to nanometric vesicles together with vesicle clusters was present in RA supernatants in the presence of IL-17 and TNF-α. OA samples in the absence or presence of inflammatory cytokines showed a fluorescent background, together with bright lipid clusters. 

To visualize details at the nanometric scale, we used a transmission electron microscope (TEM) with negative staining (Figure 3(Aa)). Two percent of phosphatidic acid was used, as a negative stain that dissolves easily in the aqueous media. The intensity of the staining (more or less dark) is dependent on the viscosity of the medium (e.g., a low-viscosity medium will have more stain and thus will appear darker). This dye does not penetrate the lipid bilayers; hence, a white coloration for the hydrophobic core of the bilayers is obtained. The stain enters the aqueous layers trapped between phospholipid bilayers, allowing us to count the number of bilayers composing the phospholipid membrane of the vesicles. 

The results of the analysis confirmed the presence of micrometric vesicles in supernatants of H cells. Vesicles were well separated from each other by a dark background (Figure 3(Ad)). This background was darker than the vesicle center, which, according to the dye behavior described above, was less permeable. A zoom on a selected vesicle (Figure 3(Ad) insert) showed the presence of dark layers (stain-permeable) between white (stain-impermeable) films. This corresponded, in the shown case, to a stack of three lipid bilayers, highlighting the multilayer structure of the vesicle shell (Arrow). Thus, in the healthy samples, we highlight the presence of multilamellar vesicles (membranes from 2 to 5 lipid bilayers) similar to those observed in synovial fluids recovered from different animal models [60] and to those reproduced ex vivo and described as “gel-in” type of vesicles [49,61]. 

The analysis of OA supernatants (Figure 3(Ae) and insert) showed lipid structures very different from that of H. We observed that vesicles were surrounded by a light background. This can be explained by the presence of a high concentration of a viscous substance, most probably hyaluronic acid, not included in vesicles, as reported in the case of “gel-out” biomimetic fluids previously analyzed [1]. Of note, as mentioned above, a highly fluorescent background was observed for OA supernatants, whereas a low PLT amount was estimated. The viscous structure of “gel out” samples may explain this high fluorescence intensity (reflection in the medium with a higher reflection index) present in samples, despite rather low phospholipid amounts.

Nanometric and dispersed vesicles, were present in RA samples in non-inflammatory medium (Figure 3(Af) and insert), confirming confocal microscopy observations. They were either uni- or bilamellar. The medium present outside the vesicles had an intense dark-grey shade, suggesting that the outside medium had a low viscosity, and was highly permeable to the stain, which is typical of a “gel in” vesicle type [1,62]. 

Supernatants obtained in the presence of an inflammatory environment for H (Figure 3(Bd)), OA (Figure 3(Be)), and RA (Figure 3(Bf)) cells were devoid of well-defined multilamellar structures, and only contained vesicles with a light grey coloration in the center surrounded by a dark thin shell. This state was similar to lipid droplets with a hydrophobic core and an amphipathic monolayer shell. 

### 2.3. Mechanical and Tribological Characterization

#### 2.3.1. Tribological Properties of Supernatant in the Case of OA and RA

The ultrastructure of synovial vesicles produced by FLS was severely altered, both by the pathological state of the patient from which cells were collected and by the presence or absence of inflammatory agents. As the role of synovial vesicles in the synovial joint is a lubricating one, we tested their tribological properties.

In order to reproduce realistic conditions for a synovial joint in a boundary lubrication regime, a homemade biotribometer (Figure 4A) was used, as previously described [62]. In brief, this homemade biotribometer enables in situ fluorescence contact visualization and the measurement of the friction coefficient between a hydrophilic soft lens made of hydroxy-ethyl-methacrylate (HEMA), which is a good substitute for the cartilage, and a flat borosilicate glass plate, each covered with a single phospholipid bilayer obtained by the liposome fusion method [63]. The surfaces are immersed in the lubricant liquid; in this case, the concentrated supernatants obtained from H, OA, and RA synoviocyte cultures, in the presence or absence of IL-17 and TNF-α.

The visualization of the two lipid-covered surfaces after rubbing indicates the wear of the surfaces in contact (a) no wear is recorded when the interface is homogeneous and consistent in the viewing octagon of the microscope (b) interface degradations appear as clearer clusters in the viewing octagon of the microscope (Figure 4B). In the case of H in control, a clear and homogeneous image was obtained, attesting that there was no wear of the lipid surface (Figure 4C). In the case of OA and RA, wear was observed as a deposit of lipid clusters (Figure 4D,E). The wear for the lipid interfaces seen in the case of RA supernatant was more persistent than that of OA supernatant. The friction coefficient was measured in situ (Figure 4I, white bars) and an increase in the wear was correlated with a friction increase. For lipid interfaces immersed in OA and RA supernatants, the coefficient of friction was two times higher (Cf~0.04 and 0.046, respectively) than those immersed in H supernatant (Cf~0.02) (Figure 4I). Thus, the wear and the coefficient of friction increased significantly for supernatants issued from pathologic conditions.

In the case of an inflammatory environment, lipid interfaces in the presence of H (IL-17/TNF-α) supernatant degraded upon friction and lipid clusters appeared (Figure 4F). The coefficient of friction was significantly higher than the one obtained in control conditions (Cf~0.077) (Figure 4I, green bars). Thus, inflammation altered not only vesicle morphology but also SF lubricating performances.

In the presence of OA (IL-17/TNF-α) supernatant, the coefficient of friction was significantly higher (Cf~0.083) than in the presence of OA (CTRL) (Cf~0.04). No significant difference between those obtained in the presence of the RA (CTRL) and RA (IL17 + TNF-α) (Cf~0.045), was obtained (Figure 4I, green bars). The presence of lipid clusters for OA and RA (IL-17/TNF-α) (Figure 4G,H) indicated pronounced wear. 

To sum up, an inflammatory environment such as the combined pro-inflammatory cytokines (IL17/TNF-α) accentuated the wear and increased the coefficient of friction, for H, and to a lesser extent for OA samples, but not for RA samples.

#### 2.3.2. Mechanical Properties of Synovial Vesicles Evaluated by Atomic Force Microscopy

The biomechanical behavior under stress of the isolated vesicles produced by different synoviocyte cultures (H, OA, and RA) under both inflammatory and non-inflammatory environments was performed by AFM indentation tests using a spherical-tipped cantilever, as previously described [64]. 

#### 2.3.3. Morphology of Synovial Vesicles by Atomic Force Microscopy

**Figure 5:**
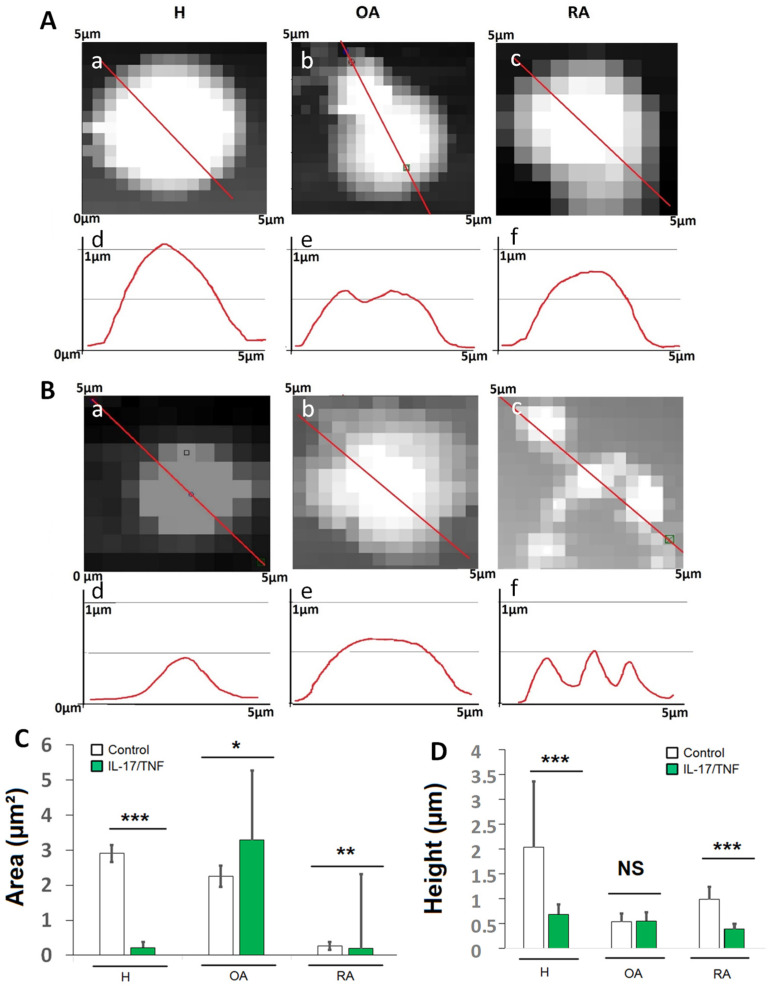
Characterization of vesicles from supernatants of healthy and pathological (OA and RA) FLS in the absence or presence of inflammatory environment (IL-17: 50 ng/mL, TNF-α: 1 ng/mL). Vesicle sectional curves and height topographies under standard (**A**) and inflammatory conditions (**B**). Differences in the height topographies (**a**–**c**) and sectional curves (**d**–**f**) depending on culture conditions were observed by Atomic Force Microscopy. Average vesicle area (**C**) and average height (**D**) as a function of pathology and inflammatory environment compared to control and represented by mean ± SEM and with ANOVA test *** *p* < 0.0001, ** *p* < 0.001 * *p* < 0.01 and NS non-significant.

In the absence of inflammatory cytokines, before indentation, the height topographies and sectional curves of immobilized vesicles obtained from the different culture conditions were analyzed (Figure 5). H (CTRL) vesicles were spherical with micrometric size (Figure 5(Aa,Ad)). An average cross-sectional vesicle area of ~3 µm^2^ and an average height of 2 µm (Figure 5C,D) were calculated, after appropriate corrections [64]. 

Sectional curves and height topographies of OA (CTRL) vesicles (Figure 5(Ab,Ae)) showed small vesicles with a strong tendency to aggregate, leading to the formation of clusters. In AFM observations, each cluster reacted as one vesicle, giving an average area of approximately 2 µm^2^ (Figure 5C), whereas the average height was around 0.5 µm (Figure 5D). RA (CTRL) vesicles were submicrometric and well separated from each other (Figure 5(Ac,Af)) with an average area of 0.7 µm and 0.2 µm, respectively (Figure 5C,D). 

The addition of combined pro-inflammatory cytokines (IL-17/TNF-α) to H cells reduced vesicle size (Figure 5(Ba,Bd)) with an average area less than 0.5 µm^2^ and a height near 0.5 µm (Figure 5C,D).

A light grey shadow was observed around OA and RA vesicles in the presence of inflammatory agents, revealing the presence of a viscous substance outside the vesicles (Figure 5(Bc,Bd)). We can relate this observation to confocal micrographs (Figure 3), which showed a strong background intensity around OA (IL-17/TNF-α) and RA (IL-17/TNF-α) vesicles, and argue that OA and RA vesicles are surrounded by a polymer gel. For OA (IL-17/TNF-α), the height of vesicles remained unchanged with respect to OA under control conditions, but a significant increase in the average area of clusters was recorded, which attained over 3 µm^2^ (Figure 5C,D). RA (IL-17/TNF-α) vesicles showed a tendency to aggregate and a significant decrease in the height and in the average area compared to RA vesicles (Figure 5C,D).

Overall, AFM observations confirmed the presence of regular spherical-shaped synovial vesicles in the healthy medium. This structure was altered in OA and RA samples, and under the effect of IL-17/TNF-α, towards smaller vesicles with a gel-out structure. In OA and in inflammatory RA samples, these vesicles have a tendency to aggregate. Such transformations may be responsible for the lower lubricant capacities of SF as a whole (increase in friction and wear). 

### 2.4. Response to Mechanical Stress at the Scale of a Single Vesicle

**Figure 6:**
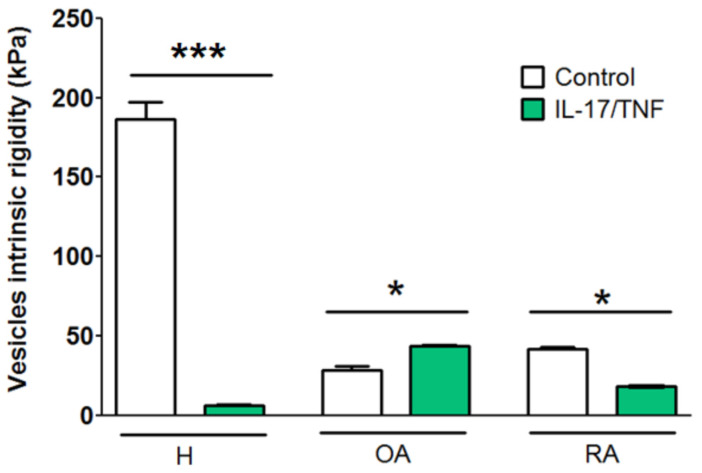
Mechanical characterization of vesicles in supernatant by Atomic Force Microscopy in healthy (H) and pathological conditions (OA and RA) in the absence or presence of inflammatory environment (IL-17: 50 ng/mL, TNF-α: 1 ng/mL). Vesicle intrinsic rigidity as a function of pathology and inflammatory environment compared to control and represented by mean ± SEM. * *p* < 0.01 and *** *p* < 0.001.

We tested the single vesicle response to mechanical stress and determined the vesicle intrinsic rigidity by AFM indentation. As shown in the histograms in Figure 6, contrary to the very rigid H control vesicles (~200 kPa), vesicle intrinsic rigidity decreased to 40 kPa in the OA- and RA-derived vesicles. Among the pathological systems, RA vesicles were more rigid than OA vesicles (Figure 6, white bars).

Under inflammatory conditions (Figure 6, green bars), H vesicles massively lost their intrinsic rigidity (≤10 kPa). However, the intrinsic rigidity of OA (IL-17/TNF-α) vesicles increased significantly compared to OA (CTRL) vesicles, while RA (IL-17/TNF-α) vesicles decreased significantly compared to RA (CTRL) vesicles. Yet, under all conditions, the intrinsic rigidity of the synovial vesicles remained lower than 40 kPa, which is substantially lower than the rigidity of healthy synovial vesicles.

## 3. Discussion

The synovial fluid is a highly viscous biofluid that plays a crucial role in joint motion with its tribological properties that allow minimal wear and friction [65]. The different components of the SF adopt organizations such as synovial vesicles to ensure lubrication of cartilaginous surfaces and to facilitate the absorption of mechanical stresses during a normal gait cycle or in case of shocks [66]. 

In a previous study, we showed that structural and tribological properties of the SF are altered in the case of OA and RA pathologies [49]. In OA, the synovial membrane is characterized by increased fibrosis, vascularization, and immune cell infiltration, as well as the production of inflammatory factors and neuropeptides [9], which may in turn affect FLS physiology. These changes contribute to OA pain. In RA, inflammation, secondary osteoarthritis, as well as central and peripheral sensitization, play important roles in the origin of pain [11]. A recent review also points out a close association between apoptotic dysregulations, an increased number of FLS and inflammatory cells, and increased central hypersensitivity in various types of chronic and neuropathic pain in RA [12]. Consequently, both pathologies involve FLS dysfunction. However, the relationship between such physiological imbalance, FLS function, and defects in lubrication has been little explored.

The aim of the present study was to assess if these perturbations in SF from OA and RA patients were due/associated with an aberrant synthesis of synovial vesicles coming from FLS and to assess the role of inflammatory factors in the loss of lubricating capacities. 

In a first step, we established an in vitro cell culture model of synovial fluid FLS EV synthesis, using healthy FLS as a control, osteoarthritis FLS as a model for degenerative pathologies, and rheumatoid arthritis as an inflammatory autoimmune pathology. 

Pathological state (OA or RA) severely impacted synoviocyte morphology. Moreover, inflammatory conditions had a drastic effect on the morphology of H control, while their effect was less pronounced, although observable, for OA and RA, going towards a more elongated form for OA cells and smaller cells for RA. The subsidiary question was whether such modifications would equally alter the quality of the synthesized vesicles and eventually the lubricating properties of SF.

Collectively, our data indicate that FLS are subject to significant changes in cell morphology and in vesicle distribution inside cells in RA and OA pathologies (Figure 2). In addition to a modified distribution within the cell, the synovial vesicles from OA and RA supernatants also have a different morphology as attested by confocal microscopy, TEM, and AFM characterization. The three techniques comforted the finding that H-type synovial vesicles are large microvesicles with a strong contrast between the vesicle core and a black extravesicular background (Figure 3 and Figure 5). We can conclude that H vesicles correspond to a gel-in configuration where the core of the vesicle is viscous and the external medium is more fluid. This observation is in line with those reported on SF samples obtained from healthy volunteers [49] and from various animal sources [60]. An organization of synovial fluid as a microvesicle-included gel is associated with a good lubricating function, attributed to a ball-bearing effect, offering excellent tribological properties to the lubricant film. We have previously proposed this phenotype of synovial vesicles as a hallmark of a healthy, well-functioning synovial fluid [1,49,60].

Pathological situations such as RA and OA did not stop vesicle synthesis (RA phospholipid content in the supernatant even increased) but they severely altered vesicle size and structure. 

Supernatants from OA synoviocyte cultures revealed the presence of numerous vesicle clusters, a high fluorescence background, but low PLT concentrations (Figure 3). We can tentatively explain the lack of correlation between the high fluorescence supposing the presence of lipid membranes and the low PLT concentration by (i) a poor solubilization of the clusters in solvents used for lipid extraction or (ii) the presence of a viscous structure outside vesicles which may artificially increase the fluorescence intensity of the background (reflection in the medium with a higher reflection index). The analysis of OA supernatants by TEM and AFM supports the latter. Vesicles were surrounded by a light background, which can be explained by the presence of a high concentration of a viscous substance, most probably hyaluronic acid, not included in vesicles. This corresponded to a gel-out status [61], which generally resulted in poor lubricating and mechanical properties of synovial fluid. Small-sized vesicles (20 to 180 nm) were present in supernatants from patients with RA pathology.

The consequences in lubricating performances are dramatic, resulting in high wear and high friction coefficients in the presence of OA and RA supernatant as lubricant. This can be explained at the scale of the individual vesicles: both RA and OA vesicles have up to ten-fold lower intrinsic rigidity than H vesicles (Figure 6) as measured by AFM. The above-mentioned ball-bearing effect of synovial vesicles is therefore less effective to ensure a good lubricating function. 

We can safely conclude that OA and RA pathologies alter intrinsic vesicle synthesis by FLS. Are such characteristics the result of an inflammatory situation in the joint or are they intrinsically related to cellular modifications reported in Figure 2?

To answer that question, an inflammation-mimicking medium (IL-17: 50 ng/mL, TNF-α: 1 ng/mL) was added to H, OA, and RA cell cultures. The addition of combined pro-inflammatory cytokines (IL17 + TNF-α) modifies the morphology of healthy FLS, as well as the intracellular distribution of vesicles to be released, with a high vesicle density in the areas of intercellular connections. 

Drastic modifications are also observed in the shape, structure, and lubricating performances of the released synovial vesicles. Surfaces immersed in H-inflammatory supernatants show significant wear and a high coefficient of friction (Figure 5), whereas the intrinsic rigidity of the vesicles is drastically reduced (Figure 6). 

The inflammatory environment had a less drastic effect on OA or RA. RA is an inflammatory disease and we can suggest that inflammatory elements are already present in the medium. RA vesicles are small, have the appearance of lipid droplets, and are surrounded by a gel viscous medium. This result is in line with the significant decrease in phospholipid percentages in SF from RA patients concomitant with a strong increase in neutral lipid content previously described [49].

Altogether, synoviocyte culture models are a good system for the analysis of synovial fluid in healthy and pathologic joint and have the advantage to decouple synovial vesicle synthesis from the influence of the plasma exudate. An altered vesicle synthesis is intrinsic to the pathological cell and structural alterations are observed at the cell and vesicle level. Moreover, an inflammatory environment may induce cell dysfunction, which leads to a pathological phenotype of vesicles released.

Two parameters directly correlate with good lubricating and nanomechanical properties: (i) the occurrence of large multilamellar synovial vesicles, and (ii) a microvesicle gel-in status. By contrast, nano-vesicles and a gel-out status are associated with poor lubricating, nanomechanical properties, and deterioration of the lipid bilayer interface upon friction tests. The latter profile is observed in synovial fluids collected from patients with inflammatory joint disease, e.g., RA, and supplemented with pro-inflammatory cytokines. Synovial fluids from degenerative joint diseases, e.g., OA, show an intermediate profile. EV released by FLS in the SF may therefore constitute an interesting biomarker for diagnostic and prognostic of joint pathologies. In turn, extracellular synovial vesicles should be taken into consideration to design therapeutic substitutes for synovial fluids and in the assessment of prosthetic implant characteristics. 

## 4. Materials and Method

### 4.1. Biological Samples and Ethic Statements

FLS were obtained from the synovial tissue of nine patients undergoing joint surgery, among which three patients fulfilled the American College of Rheumatology criteria for rheumatoid arthritis, and three patients for osteoarthritis. RA and OA patients all presented severe pathological states requiring hip prosthesis implants. The remaining three patients defined below as healthy (H) were patients undergoing surgery for mechanical issues (ligament rupture). Each individual signed an informed consent form and the protocol was approved by the Lyon teaching Hospitals review board (number AC-2016-27-29) according to French Public Health laws (art R1243-57, art L1121-1-1, art L 1121-1-2). All methods were performed in accordance with the relevant guidelines and regulations. Patients were anonymously selected in terms of gender and age within the framework of the clinical protocol.

### 4.2. Cell Culture Conditions

As previously described [67], synovial tissue was minced into small pieces which were allowed to adhere to 6-well plates in Dulbecco’s Modified Eagle’s Medium DMEM, supplemented with 10% (*w*/*v*) fetal bovine serum (FBS; Gibco, Grand Island, NY, USA), 2% (*w*/*v*) penicillin/streptomycin, 1% (*w*/*v*) L-glutamine, amphotericin B (all purchased from Eurobio Scientific, Les Ulis, France), and plasmocyn (InvivoGen, Toulouse, France). FLS grew out of the tissue and colonized the plastic dishes until reaching confluence. FLS were then trypsinized and grown in 75 cm^2^ cell culture flasks in a humidified atmosphere (5% *v*/*v* CO_2_) at 37 °C. Growth medium (30 mL) was replaced twice a week, and when the cells were confluent they were trypsinized and 150,000 cells were transferred in 75 cm^2^ cell culture flasks in 30 mL supplemented DMEM for continued growth. 

The cells in this study were used at passage 6 (P6), unless otherwise mentioned. Once P6 attained, the medium was not changed but was supplemented each week with 5 mL of fresh DMEM supplemented as described above. Cell viability was checked with the live-stream image method as previously described [67] and was found to be over 90% for the duration of the analysis.

For analysis under inflammatory conditions, 50 ng/mL IL-17 (Dendritics, Lyon, France) and 1 ng/mL TNF-α (R&D Systems, Lille, France) were added to the medium at passage 6. From this point, the medium was not changed, but each week 5 mL of fresh supplemented DMEM containing 50 ng/mL IL-17 and 1 ng/mL TNF-α was added to the flasks. Cells were incubated continuously in presence of these inflammatory factors for 20 days. Cell viability was not affected by the presence of inflammatory factors.

For EV analysis, the cell culture medium was collected after 20 days, unless otherwise indicated. 

### 4.3. Visualization of Cells and Synovial Vesicles by Confocal Microscopy

At the indicated passage and time point, cells were stained with a lipid membrane fluorescent marker Dil (1,1′-dioctadecyl-3,3,3′,3′-tetramethylindocarbocyanine Perchlorate, Thermo Fisher Scientific, Waltham, MA, USA) for confocal microscope observation (Zeiss Axio Examiner Microscope with an LSM 700 confocal head, Zeiss, Oberkochen, Allemagne). 

The supernatant was removed and cells were washed three times with PBS. Then, 2 mL of cell culture medium containing 5 µl of commercial DiI solution was added to the flasks and incubated for 15 min. Flasks were rinsed again to remove excess stain. Visualization was done in PBS, with a X5 objective and then a X63 objective, using a 550 nm laser scan. The same acquisition parameters were used for all experimental conditions for a good comparison of fluorescence intensity and to ensure that the intensity was related to the quantity of the existent lipid membranes. For each flask, three regions of interest were delimitated and three images were taken in each image. Three flasks were prepared for each condition. The option “Enhance Contrast–saturated pixel 0.4% Normalize and Equalize histogram” was used. Cell area was determined using ImageJ win 64 software (National Institutes of Health, Bethesda, MD, USA) [68]. In brief, the cell surface was delimitated manually using the polygon selection contour function and the surface was estimated with the function Analyse/Measure. Elongation was calculated for the same cell as the ratio between the maximum and the minimum diameter of the cell. For each condition, 20 cells were analyzed. Images were recorded at an intermediate zoom to have a sufficient number of cells in the field (at least five cells per image).

For visualization of vesicles present in supernatants, 2 µl of the commercial DiI solution was added to 1 mL of supernatant and incubated for 15 min at 37 °C. About 10 µl of the labeled supernatant was introduced between two glass slides (cleaned with a mixt of sodium bicarbonate and ethanol). The distance between glasses was fixed at 0.1 mm with adhesives tapes. Three images were taken with a X63 objective using the same scan laser 550 nm and the same acquisition parameters were used for accurate comparison between all experiments. Vesicle number and size were estimated with ImageJ software using an automatic threshold (Ajust/Treshold function) calculated over a dark background chosen manually. The “analyse particles” option was used to count the vesicles present in the image and to obtain an estimation of their size. Considering the resolution of the images, this is a rough estimation of the vesicle size limits. For each condition, 10 images were analyzed and they contained at least 30 vesicles.

### 4.4. Vesicle Visualization by Transmission Electron Microscopy

Vesicles in supernatants were visualized by transmission electron microscopy (TEM) using the MET JEOL 2100F instrument (Jeol Ltd., Akishima, Japan) with the negative staining technique as previously described in [60]. In brief, a volume of 100 µL of each supernatant was conserved with 100 µl of dimethylsulfoxyde (DMSO) at −35 °C (Merck, Saint-Quentin-Fallavier, France). For visualization, samples were diluted four times to separate vesicles and decrease viscosity to obtain a better contrast in TEM. Then, samples were deposited on a carbon-coated grid and then stained using 2% phosphatidic acid diluted in HEPES buffer pH 7.4 (Merck, Saint-Quentin-Fallavier, France). 

### 4.5. Determination of Phospholipid Concentration Using Stewart Assay

To determine the concentration of total phospholipids (PLT) in each supernatant (H, OA, and RA) in the absence or presence of a pro-inflammatory environment, 1 mL from each supernatant was collected and lipids were extracted using the Folch method [69] with a mix of chloroform and ethanol (2:1, *v*:*v*). Phospholipid content was measured with the Stewart method [70]. 

### 4.6. Tribological Tests

To analyze the tribological properties of synovial fluid, 30 mL of each supernatant (H, OA, and RA) in the absence or presence of a pro-inflammatory environment (IL17 + TNF-α), were concentrated by ultracentrifugation to 1 mL of concentrated lubricant and tested on our home-made bio-tribometer, as previously described [62]. Briefly, this device allows one to simultaneously measure the friction coefficient between a hydrophilic soft lens made of hydroxyl-ethyl-methacrylate (HEMA) hydrogel (Corneal, Metz, France) and a flat borosilicate glass plate, both covered with one fluorescent lipid bilayer (obtained by fusion of POPC liposomes doped with 1% NBD-PC) and immersed in 1 mL of concentrated supernatant of different FLS cultures. The lipid bilayer was visualized with confocal microscopy before and after rubbing the two plates to estimate wear [62]. As previously shown, this assembly represents a realistic bio-tribological model of the synovial fluid/cartilage interface of synovial joints [63,71].

An average pressure of 0.3 MPa was imposed, resulting in a contact area diameter of about 2 mm [72]. These parameters guarantee a good observation by fluorescence microscopy of the contact surfaces after sliding. The friction coefficient was calculated as the ratio between the tangential force and the normal load [72]. 

### 4.7. Analysis of the Intrinsic Rigidity of Vesicles by Atomic Force Microscopy (AFM)

A biotin–streptavidin coupling was used to immobilize vesicles from different supernatants on glass slides for mechanical characterizations, with a protocol adapted from [73]. PE-biotin was incorporated into the vesicles by gentle shaking under a rotary system. Streptavidin was coated on glass slides by incubation for 2 h in HEPES 10 mM pH 7.4 buffer at room temperature. Glass slides were extensively rinsed with buffer after incubation. PE-biotin-containing vesicles were deposited on streptavidin-coated slides and incubated for 20 min at 37 °C, then washed with HEPES buffer to eliminate the excess vesicles.

**Figure 7:**
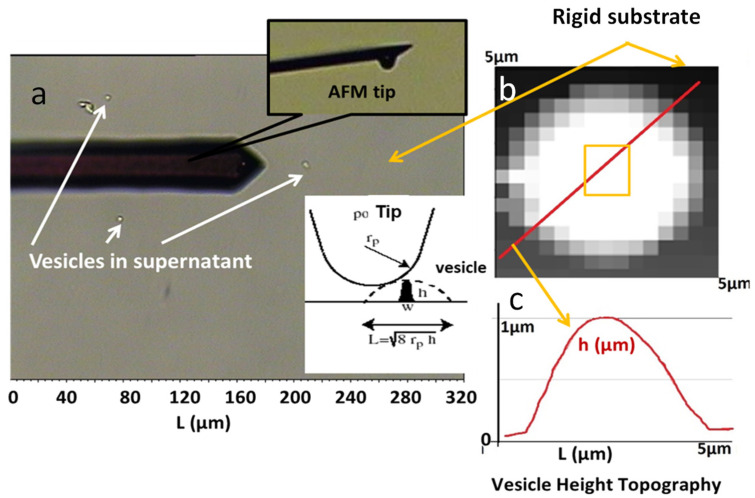
AFM settings for indentation tests performed on synovial EV. Vesicles were fixed on a glass slide through biotin–streptavidin coupling and were tested in HEPES buffer using a cantilever with a spherical tip (**a**). Insets: a zoom of the tip and a cartoon of the tip interacting with a vesicle. Vesicle height topographies (**b**) and sectional curves (**c**).

To analyze the biomechanical properties of single vesicles, we used an atomic force microscope (MFP-3D, Asylum Research, Oxford Instruments, Oxford, UK). Indentations tests were applied using the Igor Pro 6.2 software (Wave Metrics, Inc., Portland, OR, USA) as previously described in [64], with an approach-retract mode. Because the choice of the tip is crucial in AFM approach-retract tests, we used a 5 μm diameter, borosilicate spherical tip cantilever (CPPNP-BSG Colloidal probe) (Figure 7a and inset) whose spring constant of k ~0.2 N/m has been measured using the thermal noise method. This choice allowed us to have a global rigidity measurement of a vesicle due to the size of the indentation tip, which is bigger than that of the vesicle, on one hand, and to deform by about 10% of vesicle height and thus to obtain well-defined curves, on the other.

A first AFM experiment was performed in order to locate the vesicles and have their topography (Figure 7a). In this preliminary experiment, the velocity of the cantilever was fixed at 5 µm.s^−1^ and approach/retraction of the cantilever between 0 and 3 µm from the glass surface was carried out. Therefore, 20 × 20 measuring points in a square of approximately 5 × 5 µm have been recorded in order to obtain a complete image of the vesicle. The images are constituted of the ~400 force–displacements curves. Thus, we obtained non-classical topography images for which each pixel corresponds to a height (Figure 7b,c). 

The mechanical properties of the located vesicles are evaluated from 16 force measurements at each vesicle center (in a square of 0.5 × 0.5). The approach velocity of the cantilever was deliberately very low (0.5 µm.s^−1^) to avoid artefacts in liquid media measurements. 

Two slides for each type of supernatant (H, OA, RA in the presence or absence of inflammatory factors) were prepared and analyzed and three distinct vesicles were analyzed for each glass slide. This gave 100 force curves for each supernatant analyzed. 

For analysis, a fit curve power 3/2 of the Hertz model was applied, allowing the determination of the apparent elasticity modulus E* of vesicles as previously described [64]. Thereafter, the obtained value E* will be called intrinsic rigidity. A high elastic modulus corresponds to rigid vesicles, whereas a low elastic modulus corresponds to soft vesicles.

### 4.8. Statistical Analysis

Statistical analyses were performed using KaleidaGraph version 3.6 software (Synergy Software, Reading, PA, USA). Results were expressed as mean (m) ± SEM, when N > 50 and as mean (m) ± SD when N < 50. Each variable tested here was quantitative and continuous.

Normality of a group of values was checked against a theoretical normal distribution around the mean, using a single group Student *t*-test (KaleidaGraph, Student *t*-test, test value: mean value, single group). If *p* < 0.01, the distribution was considered normal. 

For each quantitative variable (cell or vesicle area, cell elongation, PLT, fluorescence intensity, intrinsic rigidity (elastic modulus), and friction coefficient) 50 to 2000 values were analyzed. Six groups were considered: H, RA, OA (CTRL), and H, RA, OA (IL-17/TNF-α) and analyzed using an ANOVA test (KaleidaGraph, ANOVA). In case of significant differences between groups, a post hoc Tukey HSD test was used to compare groups by pairs. *** *p* < 0.0001, ** *p* < 0.001, * *p* < 0.05 and NS non-significant. In the case of cell culture kinetics, quantitative variables PLT and number of vesicles were analyzed. Five groups each of 50 values were analyzed with ANOVA and were found significantly different. The post hoc Tukey HSD test was used to compare groups by pairs. *** *p* < 0.0001 and NS non-significant.

## Data Availability

The data presented in this study are available on request from the corresponding author.
