# Correction: Filali et al. Morphological and Mechanical Characterization of Extracellular Vesicles and Parent Human Synoviocytes under Physiological and Inflammatory Conditions. *Int. J. Mol. Sci.* 2022, *23*, 13201

**DOI:** 10.3390/ijms27094009

**Published:** 2026-04-30

**Authors:** Samira Filali, Nesrine Darragi-Raies, Layth Ben-Trad, Agnès Piednoir, Saw-See Hong, Fabrice Pirot, Ahmed Landoulsi, Agnès Girard-Egrot, Thierry Granjon, Ofelia Maniti, Pierre Miossec, Ana-Maria Trunfio-Sfarghiu

**Affiliations:** 1 Immunogenomics and Inflammation Research Unit EA 4130, Department of Immunology and Rheumatology, Edouard Herriot Hospital, Hospices Civils de Lyon, University of Lyon, 69007 Lyon, France; 2 Laboratory of Research and Development of Industrial Galenic Pharmacy and Laboratory of Tissue Biology and Therapeutic Engineering UMR-CNRS 5305, Pharmacy Department, FRIPHARM Platform, Edouard Herriot Hospital, Hospices Civils de Lyon, University of Lyon, 69007 Lyon, France; 3 Laboratory of Contact and Structural Mechanics, University of Lyon, CNRS, INSA Lyon, UMR5259, Villeurbanne, 69100 Lyon, France; darraginesrine@outlook.fr (N.D.-R.);; 4 Laboratory of Risques Liés aux Stress Environnementaux: Lutte et Prévention, Faculty of Sciences of Bizerte, Université of Carthage, Zarzouna 1054, Tunisia; 5 Institute de Chimie et Biochimie Moléculaires et Supramoléculaires, ICBMS, UMR 5246 CNRS, University of Lyon, 69622 Lyon, Franceofelia.maniti@univ-lyon1.fr (O.M.); 6 Institut Multidisciplinaire de Biochimie des Lipides, 69621 Villeurbanne, France; 7 ILM, UMR 5506 CNRS, University of Lyon, 69621 Villeurbanne, France; 8 UMR 754 UCBL-INRA-EPHE, Unit of Viral Infections and Comparative Pathology, 69366 Lyon, France

In the original publication [1], there was a mistake in Figure 1 as published. The original figure contains two duplicate figures, initially intended to show that the patients who were tested in the kinetic part (part A of the figure) and those who were tested in the cell passage part (part B of the figure) are the same. However, this duplication caused an error because P6 should be set at 20 days and not 10 days as shown in the original image. The corrected Figure 1 appears below. The authors state that the scientific conclusions are unaffected. This correction was approved by the Academic Editor. The original publication has also been updated.

## Figures and Tables

**Figure 1:**
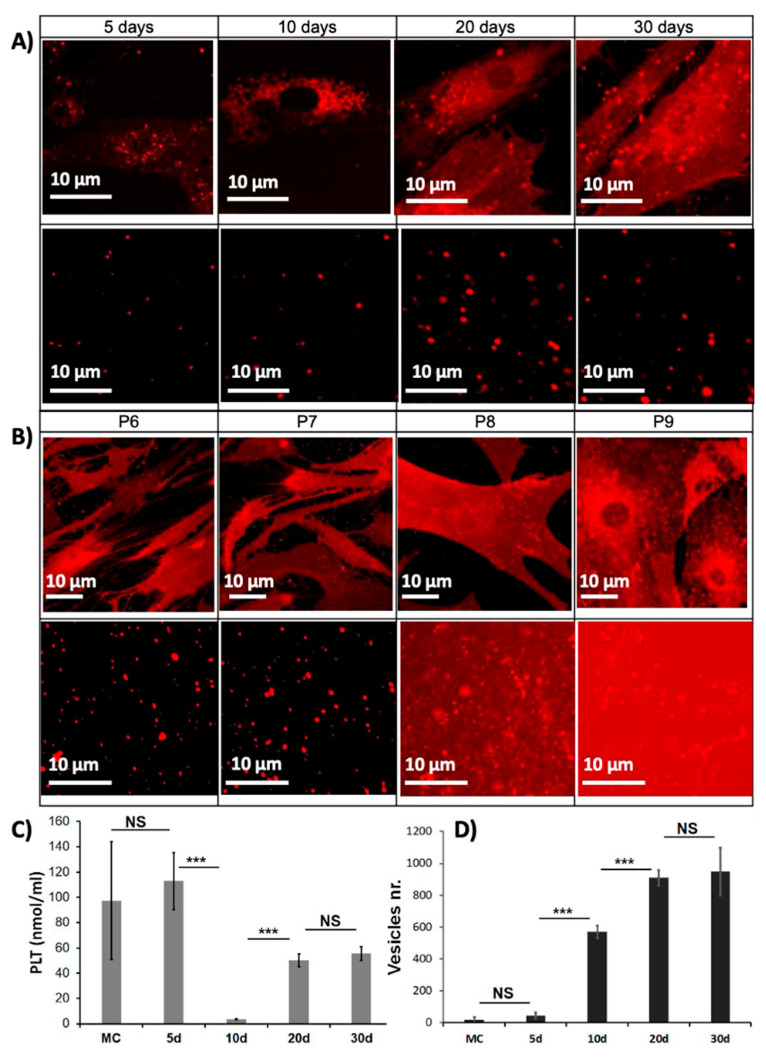
Selection of the most appropriate conditions for the structural and mechanical characterization. (**A**) Cells (**top**) and supernatants (**bottom**) confocal micrographs at 5, 10, 20, and 30 days of cell culture (X63 immersion oil microscope objective) after passage 6. (**B**) Cells (**top**) and supernatants (**bottom**) micrographs from passage 6 to 9 each after 20 days. Labeling of the different components was done by lipophilic fluorescent label “DiI” (λex~549 nm). (**C**) PLT concentration (µg/mL) in the supernatant and (**D**) number of vesicles per image as a function of cell culture day. MC, medium of culture. Bars represent mean ± SD. *** *p* < 0.0001, and NS non-significant.
